# ION-Sim: A Novel Open-Source Simulation Framework for Intraoperative Neurophysiological Monitoring

**DOI:** 10.3390/brainsci16070680

**Published:** 2026-06-28

**Authors:** Rosmary Blanco, Riccardo Budai

**Affiliations:** 1 Sano Centre for Computational Medicine, 30-054 Krakow, Poland; rosmary.blanco@gmail.com; 2 Amsterdam UMC Location University of Amsterdam, 1012 WP Amsterdam, The Netherlands; 3 University Hospital S.M. della Misericordia, 33100 Udine, Italy

**Keywords:** medical education, simulation, intraoperative neurophysiological monitoring (IONM), computational neuroscience, somatosensory-evoked potentials (SSEP), motor-evoked potentials (MEP), visual-evoked potential (VEP), compound action potential (CAP), cortico-cortical-evoked potentials (CCEP)

## Abstract

**Highlights:**

**What are the main findings?**

- This study presents ION-Sim, a novel, open-source framework designed to simulate intraoperative neurophysiological monitoring (IONM) signals and complex clinical scenarios for educational purposes.
- The framework integrates multiple physiological modules (including EEG, EMG, MEP, and SEP) with an advanced Learning Manager capable of dynamically injecting targeted clinical anomalies.
- ION-Sim is featured with a Tutor modality to interact with surgical scenarios by modifying their characteristics or creating new ones tailored to specific learning goals.

**What are the implications of the main findings?**

- ION-Sim facilitates the supervised learning of intraoperative neurophysiology through a completely hardware-agnostic approach.
- By releasing the software under an open-source license (GPLv3), the project democratizes access to high-quality neurophysiological training and facilitates the standardized assessment of skill acquisition across the global clinical community.

**Abstract:**

The educational pathway for expertise in intraoperative neurophysiological monitoring (IONM) is complex and lengthy, requiring a solid foundation in neuroscience, neurophysiology, and neuroanatomy. It also demands direct familiarity with a broad range of neurosurgical scenarios, including supratentorial, infratentorial, and spinal procedures, gained through exposure to at least ten distinct surgical approaches. Intraoperative neurophysiology must be tailored to each patient’s preoperative assessments. It relies on a variety of methods to collect, analyze, and report neurophysiological signals that are relevant to the surgical procedure. Despite its importance, there remains a substantial shortage of training tools designed to support realistic practice and skill development. To address this gap, we developed a comprehensive framework (ION-Sim) that integrates all laboratory testing modalities and adapts them to the operating room environment. ION_sim supports the simulation and analysis of spontaneous EEG and EMG activity, a wide range of evoked potentials, and intraoperative stimulus–response testing protocols. The framework provides a unified environment for practicing, testing, and validating the core neurophysiological procedures employed during neurosurgical interventions. In addition, it incorporates a robust data-management architecture, maintaining a database with system setups, user profiles, educational performance metrics, and automatically generating reports. This structure enables the longitudinal tracking of objective skill acquisition and facilitates standardized assessments of trainee progress. ION_Sim is distributed both as a ready-to-use application, suitable for direct integration into teaching and training programs, and as a modular scientific library. Through its dedicated APIs, users can design customized configurations, create novel simulation scenarios, and extend the platform to support additional research or educational objectives. It is available upon request for educational purposes and is open-source and released under the GNU General Public License, ensuring transparency, reproducibility, and long-term accessibility for the scientific and clinical communities.

## 1. Introduction

Training in clinical neurophysiology is inherently complex, necessitating the acquisition of practical skills through the execution of specific tests in clinical settings. Traditionally, this proficiency is attained during supervised rotations at accredited training centers, such as those affiliated with the Italian Society of Clinical Neurophysiology and its dedicated intraoperative neurophysiology section. However, direct learning in the operating room is not always feasible due to organizational constraints; furthermore, it occurs in a high-pressure environment that carries inherent risks. The learning process is further challenged by the heterogeneity of clinical scenarios. Although standardized monitoring protocols exist, patient-specific adaptations frequently render the educational experience unpredictable. Mastering this field requires a comprehensive understanding of neurophysiology, pharmacology—particularly anesthesiology—and cranial and spinal surgical techniques.

Currently, intraoperative monitoring simulations are largely restricted to proprietary tools bundled with specific commercial equipment. While a substantial body of literature [1,2,3] details operating room methodologies, these theoretical resources lack practical interactivity. Moreover, existing open-source and virtual reality simulators [Appendix B] focus almost exclusively on surgical techniques, omitting neurophysiological integration. To address these educational gaps, this project introduces interactive simulations encompassing both individual neurophysiological tests and comprehensive operative scenarios. Specifically, in this paper, we present ION-Sim: a novel, open-source, and freely accessible framework designed to simulate intraoperative neurophysiological monitoring (IONM) signals and clinical scenarios for educational purposes. Herein, we detail its architecture and functionalities, demonstrating its practical utility through representative case studies.

## 2. Materials and Methods

The architecture of ION-Sim is centered around a core database module designed to manage user authentication, track learning and work sessions, and archive performance metrics collected during simulations. Upon authentication, the system records learning outcomes, specifically, user responses to interactive queries, and correlates them with the active session. Ultimately, a comprehensive report is generated to consolidate all data derived from the specific simulation tests.

The pedagogical workflow begins with interactive tutorials that explain the features of the selected module, followed by active engagement with the simulation itself. Users can choose between a self-directed, in-depth study of individual modules or a guided learning pathway. The self-directed approach allows students to explore all available neurophysiological investigation methodologies, which serve as “baseline” data for surgical interventions and are essential for navigating complex simulation scenarios. Conversely, the guided learning pathway progresses from a foundational understanding of isolated modules to their integration within specific intraoperative scenarios. For example, a scenario for a supratentorial intervention near motor areas integrates multiple modules: electroencephalography (EEG), electrocorticography (ECoG), motor-evoked potentials (MEPs) for direct cortical stimulation, somatosensory-evoked potentials (SEPs) for central sulcus localization, and free-running electromyography (EMG).

### 2.1. Software Architecture

**Figure 1:**
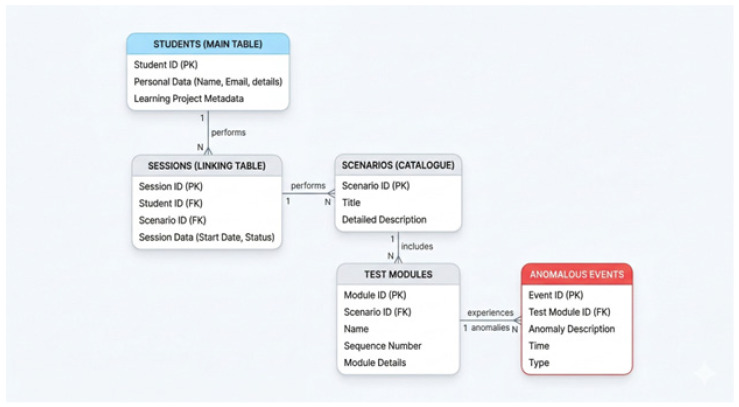
Database structure: Students (STUDENTS) participate in multiple SESSIONS, with each linked to a predefined SCENARIO from the central catalog. Each scenario contains one or more TEST MODULES, with ANOMALOUS EVENTS recorded per module as discrete or recurring patterns.

**Figure 2:**
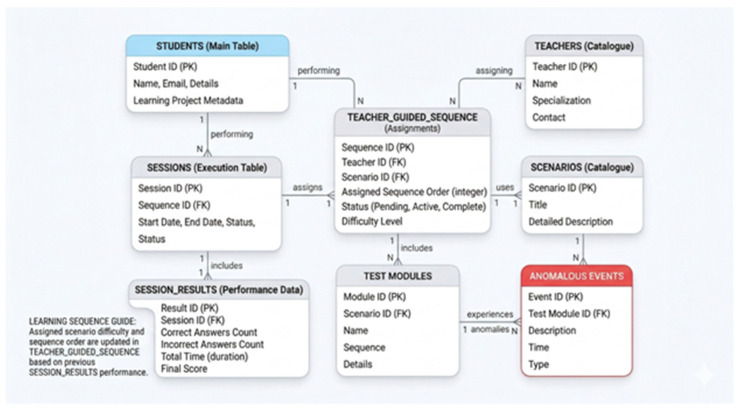
Teacher Role. The associative entity TEACHER_GUIDED_SEQUENCE connects students, teachers, and scenarios, tracking sequence status and difficulty. Teachers (TEACHERS) assign sequences to students and monitor their performance across sessions. They can also create or customize scenarios by selecting test modules and associated anomalies, enabling tailored learning paths for specific organizational or specialization needs.

As illustrated in the database schema (Figure 1), predefined scenarios specify the types of tests to be performed and include concise clinical descriptions. Each scenario is associated with multiple test modules, within which specific conditions—defined as “anomalies”—are implemented. During the initial learning phase, the system automatically selects and sequentially presents these anomalies with equal weighting. For a targeted review, users can initiate new sessions focused on specific anomaly types. The framework supports progressive learning by unlocking increasingly complex scenarios and anomalies over time. Users who achieve an “Advanced” designation can fully personalize their learning trajectory. Additionally, the system includes a Tutor role, enabling educators to design new test conditions and custom anomalies based on real-world clinical cases (Figure 2).

Building on this framework, the simulation system is designed around a centralized database repository (Figure 1). Functionally, it comprises a core Data Abstraction Layer (DAL) for database interaction (Figure 2), a suite of specialized simulation modules (Appendix A), each corresponding to a distinct physiological model, and a Learning Manager, designed to orchestrate the pedagogical progression.

### 2.2. Modules and Educational Scenario

Each simulation module follows a standard architectural pattern for interface and workflow while integrating sub-components tailored to its specific neurophysiological scenario. Upon selection from the database, simulation instances initialize automatically. A global control mechanism facilitates the dynamic injection of “anomalies” (pre-configured fault conditions retrieved directly from the repository; Figure 1).

Users can operate in two modes: (1) a self-directed, autonomous mode, allowing students to independently determine their learning objectives and choose specific modules to practice, and (2) a guided mode, seamlessly managed by the Learning Manager.

To support theoretical understanding, each module is coupled with a comprehensive knowledge base, accessible at all times through the help function, containing extended documentation on the simulation model and related neurophysiological concepts.

The primary objective of the simulation framework is to facilitate experiential learning and validate user competency within domains that require extensive practice in complex, real-world operational scenarios. The framework relies on a central database that orchestrates the entire workflow and interacts with specialized modules for simulation execution, anomaly injection, response verification, and reporting.

Upon initialization with a baseline knowledge base, users are assigned one of three roles: *Entry*, *Advanced*, or *Tutor*. Progression from an entry to an advanced level is contingent on the user’s performance metrics achieved during the initial training phase, thereby granting the user access to more sophisticated simulation functionalities: the platform challenges the student to design a personalized monitoring setup, tailored to the surgical requirements of the individual patient.

The Tutor role, assigned administratively, provides full privileges to modify pre-configured base scenarios or design novel ones, including defining specific scenario characteristics, injecting associated anomalies, and configuring the corresponding correct answers and distractors (Figure 2).

#### 2.2.1. Physiological Modules

The Physiological Modules suite comprises multiple components, each simulating specific neurophysiological activation patterns and monitoring modalities. Each module integrates an anomaly management system and a dedicated Help interface detailing specific intraoperative monitoring (IOM) procedures. These include:

- The CAP (Compound Action Potential) Simulation Module: This module simulates the stimulation of the afferent peripheral nerve trunk, with sensory fiber responses recorded at various distances from the stimulation point (e.g., wrist, elbow, axilla, Erb’s point) (Figure A7).
- The CMAP (Compound Motor Action Potential) Simulation Module: This module simulates the stimulation of the efferent peripheral nerve trunks, recording muscle responses across different segments and distances.
- The SEP (Somatosensory Evoked Potential) Simulation Module: This module involves peripheral nerve stimulation with recording montages at Erb’s point, the CV6 level, and scalp electrodes over the contralateral S1 and M1 sites. Key controllable parameters include the Stimulation Side (Right, Left), Block Average (sweep count), Overlay Percentage, Noise Amplitude (μV), and Gain Control (Figure A5).
- The VEP (Visual Evoked Potential) Simulation Module: This module simulates retinal flash stimulation with scalp recordings over ipsilateral and contralateral occipital sites. Key controllable parameters include the Stimulation Mode (Right, Left, Bilateral), the Noise Level, and the number of sweeps per block (update) (Figure A6).
- The BAEP (Brainstem Auditory Evoked Potential) Simulation Module: This module utilizes click-based acoustic stimulation with contralateral masking noise, recorded via scalp electrodes using a bilateral mastoid montage. The module features automatic identification of latency and amplitude peaks for principal wave components (Waves I, III, and V). Configurable parameters include the following: Stimulation Side (Right, Left, Alternating), Stimulus Polarity (Rarefaction, Condensation, Alternating), Stimulus Frequency (Hz), Block Average (sweep count), Overlay Percentage, Noise Amplitude (μV), and Gain Control (Figure A4).
- The MEPcb (Motor Evoked Potential-Cranio-Bulbar) Simulation Module: This module simulates transcranial scalp stimulation using a pulse train, recording from muscles innervated by cranial nerves (facial, trigeminal, glossopharyngeal, hypoglossal, and spinal accessory). It supports up to eight channels for CMAP recording and features a raster plot to track response variations over time, offering automatic marker localization for peak latency and amplitude. User-adjustable parameters include the stimulation pulse count, amplitude (%), inter-stimulus interval, and noise levels. Both stack plot and raster display scales are fully customizable (Figure A2).
- The MEPas (Motor Evoked Potential-Upper Limbs) Simulation Module: This module models muscle activation in the upper limbs following transcranial electrical pulse train stimulation, specifically targeting the first DI (first dorsal interosseous), ECD (extensor communis digitorum), and BB (biceps brachii) muscles. This module inherits the control interface and parameter definitions of the MEPcb module, presenting anomalies governed by predefined configuration schemas (Figure A2).
- The MEPai (Motor Evoked Potential-Lower Limbs) Simulation Module: This module models muscle activation in the lower limbs following transcranial electrical pulse train stimulation, targeting the FHB (flexor hallucis brevis), TA (tibialis anterior), and VL (vastus lateralis) muscles. Similarly to the upper limb module, it utilizes the standard MEP control interface and presents anomalies based on predefined configuration schemas (Figure A2).
- The D-Wave (Direct Wave) Simulation Module: This module simulates corticospinal tract activation that is recorded directly from the spinal cord. While stimulation remains transcranial, recording is performed at two distinct spinal sites: proximal and distal to the surgical intervention level. The module incorporates standard stimulation controls and features a specialized “anomaly detection” interface, which manages introduced faults and queries the user for diagnostic responses (Figure A3).
- The DCS (Direct Cortical Stimulation) Simulation Module: This module models diverse responses secondary to direct cortical stimulation, including motor responses, disruption of language function (speech arrest/aphasia), and alterations in cognitive processing (in progress).
- The CCEP (Cortico-Cortical Evoked Potentials) Simulation Module: This module simulates cortical or subcortical stimulation with the recording of evoked responses at specific cortical distances, aimed at mapping the structural connectivity of tracts and cortical areas (in progress).
- The EEG (Electroencephalography) Simulation Module: This module displays resting electrical activity and physiological variations associated with eyes open/closed states and motor activity (e.g., mu-rhythm desynchronization) and incorporates physiological cyclical variations derived from real-world clinical recordings (Figure A8).
- The ECoG (Electrocorticography) Simulation Module: This module simulates spontaneous corticographic activity, incorporating physiological cyclical variations derived from real-world clinical recordings (Figure A9).
- The EMG (Electromyography) Simulation Module: This module models spontaneous electromyographic activity, including fluctuations and voluntary contraction phenomena, typical of awake patient procedures (e.g., awake craniotomy) and different patterns of spontaneous discharges (Figure A10).
- The ANESTHESIA Simulation Module: This module displays patient metadata (age, sex, weight, height, BMI, and LBM), monitors vital signs (SpO_2_, HR, R-R interval, SDNN, RMSSD, NIBP, MAP, temperature, and RPM), and manages drug infusion via target-controlled infusion (TCI) for Sevoflurane, Propofol, Remifentanil, and Ketamine (Figure A11).

Each module implements a comprehensive mathematical and biophysical model governing signal generation at the peripheral, spinal, cortical, and muscular levels. Furthermore, this framework accounts for signal propagation dynamics along afferent and efferent pathways, alongside the simulation of cellular activation within specific neuronal nuclei of the spinal cord, thalamus, and corresponding cortical areas.

#### 2.2.2. Optimization of Evoked Response Updates

A critical performance metric in intraoperative monitoring is the temporal resolution required to acquire a statistically valid response. The system must minimize the latency between a physiological event and its visualization to promptly assess whether the signal remains aligned with the baseline or has deviated significantly in amplitude or latency.

To accelerate update rates, particularly for low-amplitude afferent-evoked potentials (e.g., SEP, BAEP, and VEP) where the signal-to-noise ratio (SNR) is inherently low, the system employs a weighted moving average (or overlapping technique) (Figure A5).

**Signal Averaging Principles:** The identification of a variation within a biological signal requires the summation of a sufficiently large ensemble of homogeneous responses (sweeps). This process relies on the principle that time-locked physiological signals are additive, whereas stochastic background noise (such as EEG or EMG artifacts) attenuates by a factor proportional to the square root of the number of trials.

While employing a weighted moving average (WMA) ensures a more rapid refresh rate for the visualized waveform, it concomitantly imposes a systemic limitation known as the ‘smoothing effect.’ By averaging incoming raw sweeps—which might reflect a newly onset acute anomaly—against a significant percentage of pre-existing data, the system inherently dampens the magnitude of sudden physiological fluctuations. As a result, the complete visualization of an abrupt signal attenuation is hindered by a mathematical lag. The true extent of the pathological event becomes fully apparent only when the continuous influx of anomalous sweeps gradually overrides the weight of the ‘healthy’ sweeps lingering in the computational buffer.

#### 2.2.3. Simulation of Spontaneous “Free-Running” Activity

The generation of spontaneous “free-running” bioelectrical activity—encompassing electroencephalography (EEG), electrocorticography (ECoG), and electromyography (EMG)—relies on the reproduction of empirical datasets derived from validated clinical recordings (Figure A8 and Figure A9). These signals are rendered using a seamless cyclic buffering technique to ensure continuous, artifact-free monitoring streams. Modality-specific anomalies are dynamically superimposed onto this baseline activity. The injection of these pathological patterns adheres to the same temporal logic and triggering criteria (e.g., onset latency, stochastic recurrence) previously defined for evoked responses.

### 2.3. Anomaly Simulation Module

The training progresses to the systematic identification of anomalies embedded within specific simulation contexts. Structurally, the Anomaly Simulation Module comprises discrete components designed to inject targeted distortions into the baseline signals generated by the Physiological Simulation Module, thereby producing pathological output waveforms.

To ensure these simulated distortions accurately reflect true surgical scenarios, the underlying algorithms are grounded in established intraoperative pathophysiology [2,3]. Furthermore, the resulting pathological patterns underwent a qualitative face validation by experienced clinical neurophysiologists, confirming that the simulated signal dynamics effectively mirror real-world intraoperative events.

Here, the students’ competencies are empirically evaluated based on their learned skills for detecting and interpreting diverse categories of simulated signal perturbations.

The system database provides these scenarios—ranging from single modalities to complex multimodal combinations—within the Scenario (catalog) table (Figure 2), which currently defines 18 distinct configurations. The architecture supports the development of additional experimental setups in the Scenario (catalog) table; however, new integrations require rigorous validation to prevent stimulus artifact overlap. The system enforces strict temporal management regarding multimodal-evoked responses, ensuring that the epoch of a new stimulus does not encroach upon the acquisition window currently occupied by another active stimulation modality.

Anomalies available for simulation are indexed within the Scenario anomaly table and mapped to specific pre-configured scenarios. The injection of these anomalies is governed by configurable parameters, including onset latency and recurrence patterns, which may be defined as singular events or periodic occurrences with fixed or randomized intervals.

The simulated anomalies are classified according to the following taxonomy:

**Amplitude Anomalies:** Percentage decrements in signal magnitude that approach or exceed established clinical warning and alarm thresholds (e.g., a 50% reduction from baseline).

**Latency Anomalies:** Prolongation of signal latency relative to baseline, indicative of slowed neural conduction velocity, reaching or surpassing predefined temporal warning limits (e.g., a 10% increase).

**Signal Quality Anomalies:** Contamination by stochastic or deterministic noise arising from diverse perioperative sources. These include electrocautery interference, suction artifacts, stimulus artifact spillovers, surgical maneuvers, and mechanical disturbances (e.g., high-speed drills or irrigation systems).

**Signal Morphology Anomalies:** Qualitative alterations in waveform configuration and spectral content. Key features include temporal dispersion, increased polyphasia, and loss of component distinctness, typically reflecting desynchronized neural volley activation.

**Technical Anomalies:** Equipment-related failures, such as recording electrode dysfunction (e.g., dislodgement or high impedance) or stimulation system faults affecting one or more modalities.

**Systemic Anomalies:** Global physiological fluctuations affecting signal integrity, induced by pharmacological agents (e.g., bolus anesthetic administration), hemodynamic instability (e.g., hypotension), or acute oxygen desaturation.

### 2.4. Learning Module

The Learning Module orchestrates the injection of anomalies into the physiological simulators and manages the subsequent anomaly interpretation tasks. This assessment is facilitated through a dedicated interface utilizing a multiple-choice paradigm, consistently presenting one correct response alongside four distractors. To comprehensively assess user proficiency, the module evaluates several key performance indicators:

**Response latency:** The time elapsed between the onset of the anomaly and the user’s initial action.

**Diagnostic accuracy:** The correctness of the selected response.

**Attempt frequency:** The number of iterations required to identify the correct solution.

**Resource utilization:** The time spent consulting the integrated “help” documentation.

Furthermore, the Learning Module features an adaptive difficulty mechanism that progressively scales task complexity based on the user’s accuracy and response immediacy. The module continuously evaluates operational behavior to foster sustained engagement and optimize the learning trajectory. Upon session completion, the framework generates a comprehensive analytical report and a final report of the user’s learning progress.

## 3. Software Implementation and Availability

**Figure A1:**
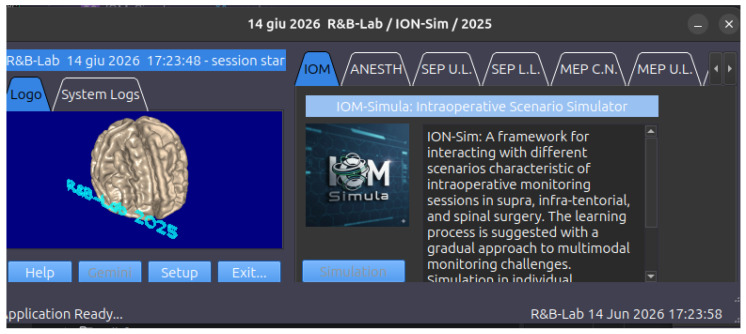
ION-Sim system home screen.

**Figure A2:**
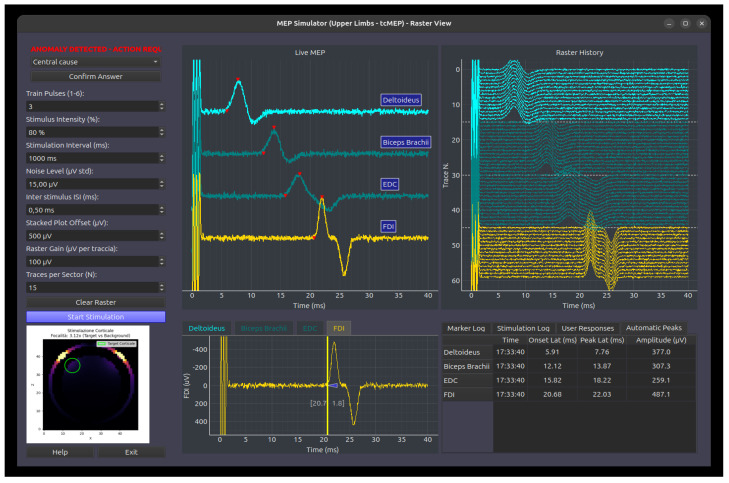
MEP module screen.

**Figure A3:**
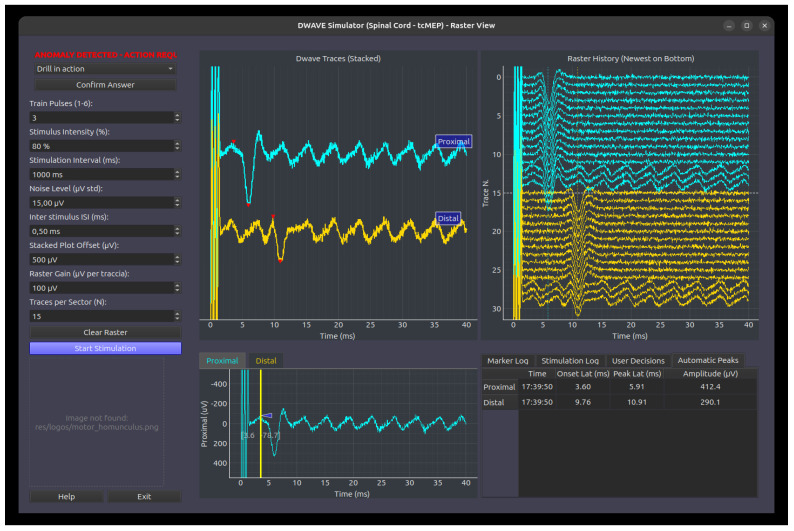
Dwave module screen.

**Figure A4:**
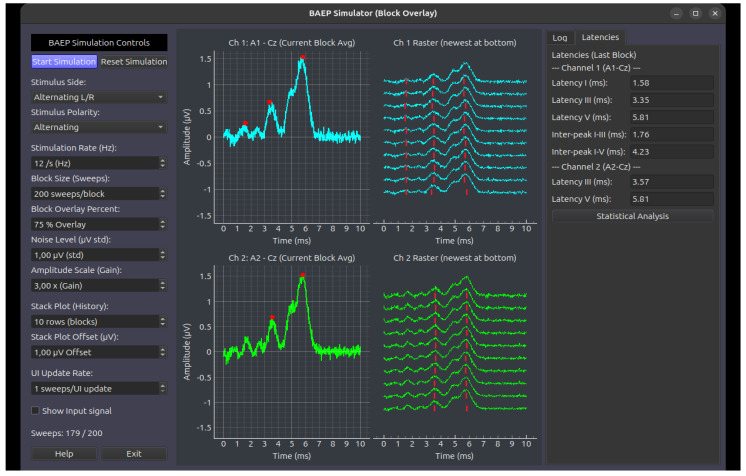
BAEP module screen.

**Figure A5:**
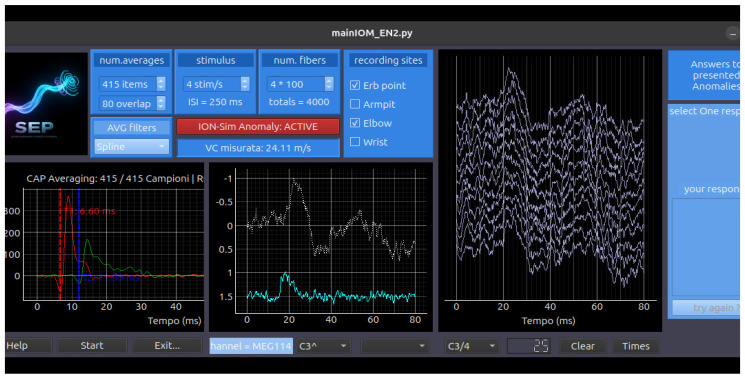
SEP module screen.

**Figure A6:**
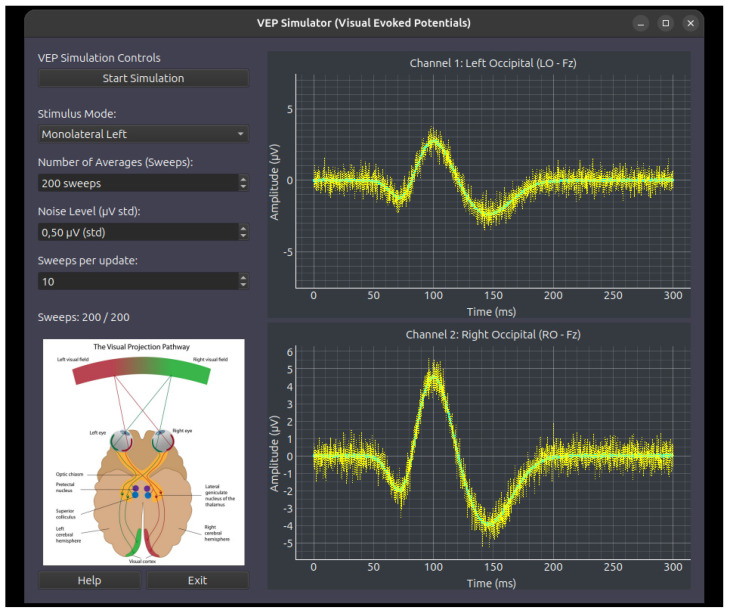
VEP module screen.

**Figure A7:**
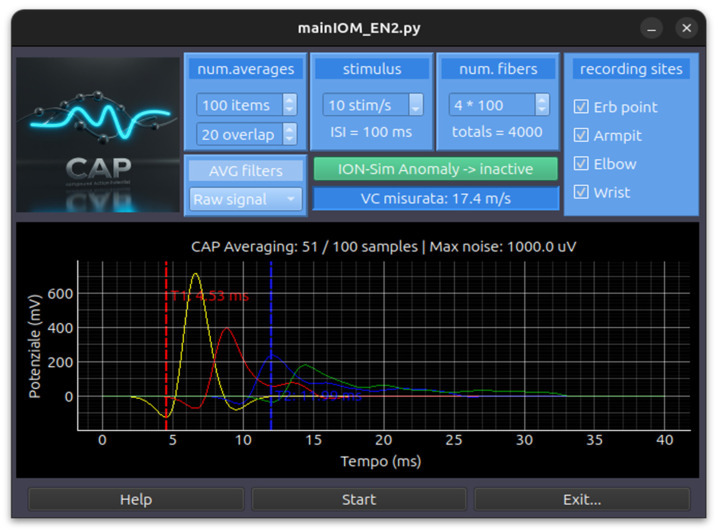
CAP module screen.

**Figure A8:**
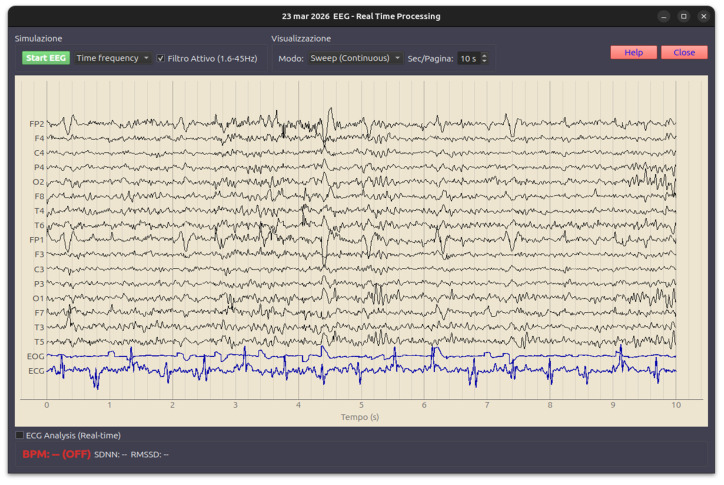
EEG module screen.

**Figure A9:**
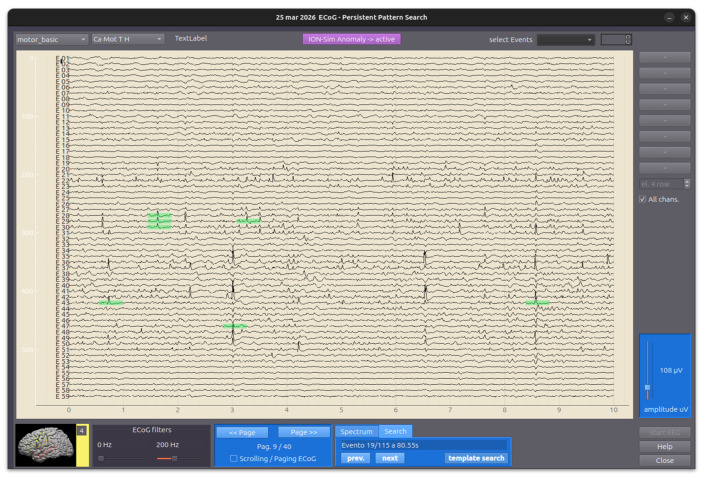
ECoG module screen.

**Figure A10:**
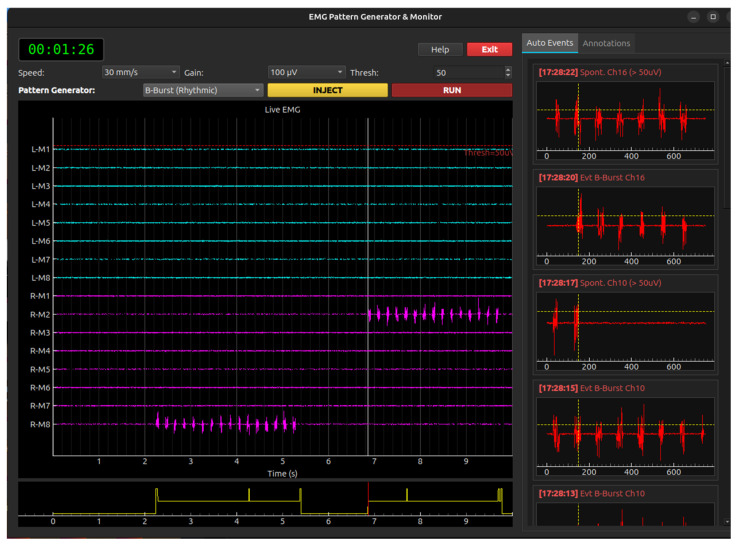
EMG free-running module screen.

**Figure A11:**
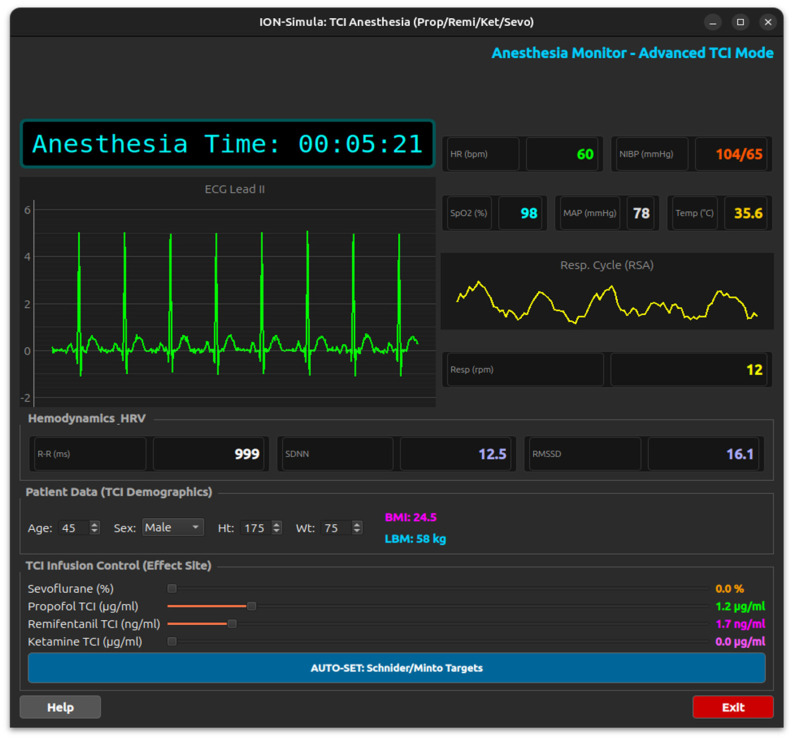
Anesthesia module screen.

The simulation framework is implemented in Python 3.12 and was chosen for its extensive ecosystem of scientific computing libraries and its robust cross-platform compatibility, released as an open-source scientific library hosted on GitHub.com. It exposes a robust set of Application Programming Interfaces (APIs) designed for seamless integration within Python or C++ development environments. The ION-Sim system home screen is shown in Figure A1.

**Technology Stack:** The architecture leverages a modular stack of specialized libraries.

**Computational Backend:** NumPy and SciPy constitute the core engine for signal processing, matrix operations, and the mathematical modeling of physiological responses.

**Graphical User Interface (GUI):** The frontend is developed using PySide6 (the official Python binding for the Qt framework), ensuring a responsive and native look-and-feel across different operating systems.

**Real-Time Visualization:** High-performance signal rendering—essential for simulating continuous EEG/EMG streams and evoked potentials—is managed by the “pyqtgraph” library, which is optimized for fast data plotting.

**User Interface Design:** The UI is engineered to streamline user interactions by abstracting the underlying database complexities. By automating session management and pre-loading scenario configurations, the system minimizes the setup burden, allowing the user to focus on the educational task.

**Licensing and Availability:** This is to foster collaboration and accessibility within the scientific community; the source code is released under the GNU General Public License v3 (GPLv3). The complete repository, including documentation and installation instructions, is publicly hosted on GitHub [https://github.com/riccardo-budai/ION_Simula Accessed on 22 May 2026].

## 4. Discussion

The landscape of simulation tools for intraoperative neuromonitoring (IONM) can be broadly categorized into commercial proprietary solutions, research-oriented platforms, and procedure-specific training systems (Table A1).

**Commercial Proprietary Solutions:** Leading biomedical device manufacturers, such as Inomed [4], Cadwell [5,6], and Neurosoft [7], provide simulation features primarily designed to familiarize users with their specific software interfaces and intraoperative settings. Among these, only Neurosoft explicitly lists a hardware-based “Patient Simulator” as a distinct accessory. However, these tools are generally closed-source and tethered to specific proprietary hardware, limiting their accessibility for broad academic or generalized training purposes.

**Research and Biomechanical Platforms:** In the open-source domain, OpenSim [8] represents a powerful standard for musculoskeletal modeling and movement analysis. While highly effective for biomechanics, its focus does not extend to the generation of specific electrophysiological signals (e.g., SSEP or MEP) required for neuromonitoring interpretation. Similarly, The Virtual Brain (TVB) [9] serves as a robust platform for simulating large-scale brain network dynamics. While it is an invaluable tool for neuroscience research, it is not designed to simulate discrete, real-time clinical scenarios for intraoperative educational applications.

**Procedure-Specific and Hybrid Systems:** Academic literature describes targeted implementations, such as a “Simulation system for intraoperative neuromonitoring” developed specifically for mastoidectomy training. This system utilizes magnetic tracking to detect the position of a probe on a physical model, generating synthetic EMG signals based on proximity to a “virtual nerve.” While this validates the clinical necessity and academic interest in such tools, it represents a hybrid solution (hardware-dependent) tailored to a single surgical procedure rather than a generalized, software-only framework.

**Surgical Procedural Simulators:** Finally, platforms like SurgeonsLab [10] and InSimo [11] offer high-fidelity neurosurgical simulations. However, these tools predominantly focus on manual procedural skills, anatomical navigation, and haptic feedback rather than on the analysis and interpretation of neurophysiological traces, which remains a distinct cognitive skill set. To contextualize the architecture of ION-Sim, it is essential to distinguish between a traditional simulator and a Digital Twin, as their objectives and operational modes differ significantly.

A **simulator** is primarily designed to study the behavior of a system under hypothetical conditions using mathematical or logical models. Its connection to physical reality is often absent or limited, relying on static or predefined input data (historical or design data). Consequently, the output is a predictive test result that does not influence a real physical asset. It serves as a powerful tool for design, “what-if” scenario testing, and offline training, operating autonomously even before the physical object exists. Conversely, a **Digital Twin** [12] aims to dynamically replicate the state, condition, and behavior of a specific, existing physical asset. It requires a bidirectional, essential connection with physical reality, which is typically established via IoT sensors.

A Digital Twin consumes real-time live data to update its internal state automatically, reflecting the exact condition of its physical counterpart. Its primary applications include real-time monitoring, predictive maintenance, and operational optimization across the asset’s lifecycle.

While ION-Sim is natively a simulation framework, its architecture is designed to support Digital Twin-like interactions through the integration of the Lab Streaming Layer (LSL) protocol. This capability transforms the framework from a closed, static system into an open, interoperable environment capable of bidirectional real-time data exchange. In the proposed architecture, the interaction with the “real world” (or external software agents) is managed through a low-latency multicast network as follows:

**Outbound Stream:** ION-Sim generates neurophysiological data and distributes it via LSL, acting as a “virtual patient”.

**External Processing (The “Twin” Loop):** This is a connected external application that captures this stream. This application can act as a dynamic “Anomaly Engine” or a feedback controller. Instead of using static parameters, it can process the incoming data and calculate perturbations or anomalies (randomized or logic-based) in real-time.

**Inbound Stream (Feedback):** The external application returns the modified parameters, or event triggers back to ION-Sim via a secondary LSL stream.

**Dynamic Update:** ION-Sim receives this control stream and instantaneously updates the ongoing simulation state. This closed-loop mechanism allows ION-Sim to function not just as a standalone trainer but as a more complex, interconnected setup.

## 5. Limitations and Future Work

The current paper primarily establishes the software architecture and theoretical biophysical modeling of the framework. It currently lacks large-scale user evaluation and quantitative data to objectively measure the platform’s impact on trainees’ knowledge acquisition. This formal pedagogical validation is deferred to future studies.

While the open architecture allows users (Tutors) to design new experimental setups and multimodal combinations, these new integrations require rigorous validation. The system relies on strict temporal management, and poorly configured custom scenarios run the risk of stimulus artifact overlap.

Currently, ION-Sim operates entirely as a software-based simulator, lacking the manual dexterity, haptic feedback, and anatomical spatial navigation offered by surgical procedural simulators. Although the framework is designed to interface with physical sensorized phantoms (e.g., mannequins) in the future to realize a true “Digital Twin,” this physical input is not part of the current implementation.

Future developments of the ION-Sim project will prioritize pedagogical validation. Having established the software architecture and theoretical modeling in the current study, the next phase involves large-scale user evaluation. As the system is being used, we will continuously collect usage and performance data from trainees. Future studies will quantitatively assess the platform’s impact on knowledge acquisition. This data-driven approach will allow us to measure the system’s effectiveness and refine the educational scenarios based on empirical feedback.

Looking forward, the framework is designed to be interfaced with physical sensorized phantoms (e.g., a mannequin equipped with cardio-respiratory and neurophysiological sensors). The phantom would provide afferent physical inputs (e.g., physical manipulation or surgical interventions by the trainee), which ION-Sim would process to generate efferent, realistic signal responses, effectively realizing a specialized Digital Twin for surgical training.

## 6. Conclusions

In this work, we presented ION-Sim, the first comprehensive, open-source framework specifically dedicated to the simulation of intraoperative neurophysiological monitoring (IONM). By decoupling simulation logic from proprietary medical hardware, ION-Sim addresses a critical educational gap in the field, offering a scalable solution to the limitations of traditional operating room apprenticeship.

Through the integration of rigorous biophysical modeling, realistic signal processing techniques, and a structured pedagogical architecture, the framework successfully replicates the complexity of intraoperative decision-making (Appendix C). The ability to simulate a wide spectrum of modalities and to inject dynamic clinical anomalies allows trainees to develop and validate their diagnostic skills in a risk-free environment.

Furthermore, the release of the software under the GNU General Public License fosters transparency and collaboration, inviting the scientific community to extend the library with new physiological models and clinical scenarios. Ultimately, ION-Sim aims to democratize access to high-quality neurophysiological training and standardized assessment metrics, thereby contributing to improved patient safety in neurosurgery. The GitHub repository is at https://github.com/riccardo-budai/ION_Simula (accessed on 22 May 2026).

## Figures and Tables

**Figure A12:**
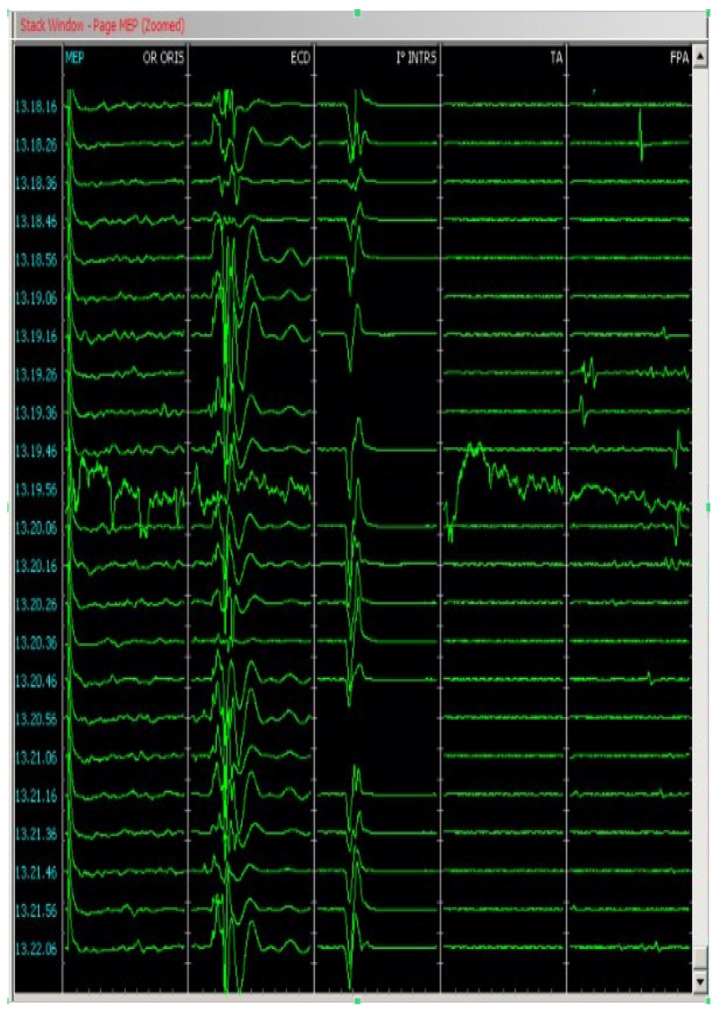
This image shows one example of real signals recorded in a surgical setting, displaying a cascade of CMAP elicited by transcranial stimulation using corkscrew electrodes.

**Figure A13:**
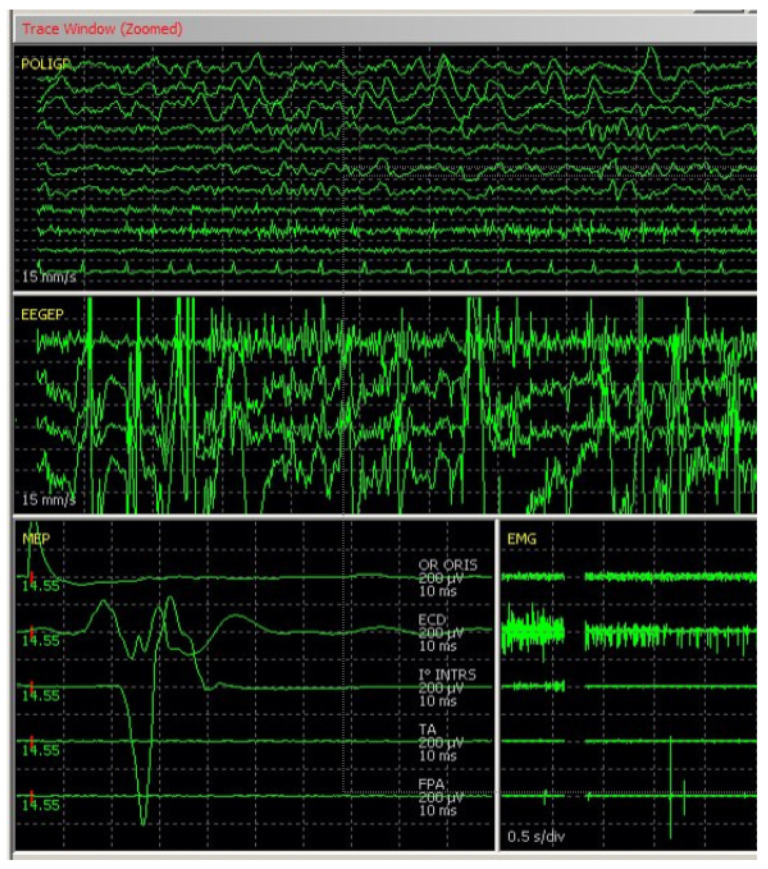
This image presents an example of a realistic multimodal scenario featuring ECoG, EEG, MEP, and free-running EMG.

## Data Availability

No new data were created or analyzed in this study. The original contributions presented in this study are included in the article. Further inquiries can be directed to the corresponding author(s).

## Abbreviations

The following abbreviations are used in this manuscript:

EEG	Electro Encephalography
ECoG	Electro Corticography
MEP	Motor-Evoked Potential
SEP	Somatosensory-Evoked Potential
VEP	Visual-Evoked Potential
Dwave	D wave
CAP	Compound Action Potential
CMAP	Compound Motor Action Potential
SFAP	Single Fiber Action Potential
MUAP	Motor Unit Action Potential
BAEP	Brain Stem Acoustic-Evoked Potential

## Appendix A

### Appendix A.1. MEP Module

The Python code implements a MEP (Motor Evoked Potentials) Simulator designed for intraoperative monitoring (IOM) training. It features a sophisticated “Raster View” and “Stacked View” to visualize multi-channel motor responses across different clinical scenarios, such as upper limbs, lower limbs, or cranial nerves.

The simulation methodology focuses on generating realistic CMAP (Compound Muscle Action Potential) waveforms that respond dynamically to stimulation parameters and clinical anomalies. Each muscle response is synthesized mathematically to reflect its physiological properties:

- Dual Gaussian Modeling: A single MEP response is created by summing two Gaussian peaks—a primary negative wave and a secondary “polyphasic” shift. This mimics the complex morphology of real muscle potential better than a single peak.
- Muscle-Specific Parameters: The simulator uses a global dictionary (ALL_MUSCLE_PARAMS) to assign unique latencies, amplitudes, and widths to each muscle. For example, the deltoideus has a short latency (8 ms), while the FPA (flexor hallucis brevis) has a long latency (37 ms), reflecting the distance from the motor cortex.

In clinical tcMEP (transcranial MEP), a single pulse is often insufficient to elicit a response due to anesthesia. The code simulates this through the following:

- Train-of-Pulses Facilitation: The simulator applies a facilitation_map where the final amplitude scale is determined by the number of pulses in the stimulator “train” (1 to 6 pulses).
- Stimulation Artifacts: It generates a series of high-frequency stimulation artifacts at the beginning of the trace, corresponding to the “Train Pulses” and “ISI” (Inter-Stimulus Interval) settings, providing a realistic visual reference for the moment of stimulation.

The simulator provides two complementary ways to analyze the signals:

- Stacked View: This displays the current MEP for each muscle vertically offset, allowing for easy identification of morphology and peak markers (onset and peak latency).
- Raster History: This implements a “waterfall” or scrolling history where the last 15 traces for each muscle are displayed. This is critical in IOM to detect subtle trends or sudden signal loss over time.

The code integrates an AnomalyInjector system as follows:

- Physical Signal Modification: When an anomaly is active, the injector modifies the physical data in real-time (e.g., causing a 50% drop in amplitude or a latency shift).
- Decision-Making Assessment: Once the user detects a change, the UI enables an “Anomaly Response” panel. The user must select the correct clinical action from a list of choices.

AI Tutor Integration: The user’s response is logged into a database and analyzed by an AI Agent. The agent provides personalized feedback and adjusts the simulation’s difficulty based on the user’s performance.

### Appendix A.2. BAEP Module

The Python code implements a BAEP (Brainstem Auditory Evoked Potential) Simulator, designed for intraoperative monitoring (IOM) training. The application uses a multithreaded architecture where a BaepWorker handles mathematical signal generation while the BaepSimulator manages the GUI and real-time visualization.

The simulation synthesizes the BAEP signal using mathematical modeling to replicate the typical waveform complex (Waves I–V) and the technical challenges of clinical recording. The core of the BAEP complex is modeled by summing multiple Gaussian peaks.

Each wave (I, II, III, IV, and V) is defined by specific latencies, amplitudes, and widths (standard deviation) as follows:

- Gaussian Model: The worker uses the formula Amp⋅e−2σ2(t−Lat)2 to generate smooth, physiologically realistic peaks.
- Anatomical Differentiation: The simulator distinguishes between two recording channels:
  ○ Channel 1 (Ipsilateral, A1-Cz) contains the full complex (waves I, II, III, IV, and V).
  ○ Channel 2 (Contralateral, A2-Cz) only simulates waves III, IV, and V, with wave III reduced in amplitude to 30%, reflecting the far-field nature of the contralateral recording.

The simulator replicates three clinical stimulation polarities that affect the morphology of the early response as follows:

- Rarefaction/Condensation: These introduce a small latency shift (0.08 ms) and control the polarity of the Cochlear Microphonic (CM).
- Alternating: The worker alternates polarity every sweep; when these are averaged, the phase-inverted CM is canceled out, which is a standard clinical technique to isolate the neural response.
- CM Generation: The CM is modeled as a sine wave that decays exponentially over time.

This mimics the process of extracting the tiny BAEP signal (often <0.5 µV) from background noise.

- Noise Injection: Every individual sweep is combined with random Gaussian noise.
- Block Averaging: Signals are processed in “blocks” (e.g., 200 sweeps).
- Overlay Method: Instead of resetting to zero after every block, the system can retain a percentage of the previous block’s data (e.g., 75% overlay). This creates a smooth “moving average” effect in the history (Stack Plot), allowing trainees to see signals evolve as noise is reduced.

The system includes an automated analysis engine using scipy.signal.find_peaks to identify waves I, III, and V. It searches for peaks within specific temporal windows (e.g., 5.0–6.5 ms for wave V) and automatically calculates inter-peak latencies (I-III and I-V), which are critical for diagnostic purposes in IOM.

### Appendix A.3. SEP Module

The Python code implements a SEP (Somatosensory Evoked Potentials) Simulator. It simulates the afferent sensory pathway from the median nerve at the wrist to the cervical spine (CV7) and the primary somatosensory cortex (S1). The application utilizes a multithreaded architecture where a CAPWorker performs the heavy mathematical calculations, and a SepSimulator (PySide6-based) handles the real-time visualization. The simulation methodology is highly sophisticated, combining biophysical modeling for peripheral signals with real-world data interpolation for cortical responses.

The Compound Action Potential (CAP) at various distances (wrist, elbow, armpit, and Erb’s point) is simulated using a bottom-up biophysical approach as follows:

- Single Fiber Modeling: Each individual nerve fiber’s action potential (SFAP) is modeled as a biphasic pulse.
- Fiber Dispersion: The simulator generates a population of fibers (default 100) with varying Conduction Velocities (CV) ranging from 20 to 65 m/s.
- Temporal Summation: The CAP at a specific distance d is the sum of SFAPs, where each fiber’s delay is calculated as Delay = VelocityDistance. As the distance increases, the individual fiber potentials “spread out” (dispersion), accurately mimicking nerve physiology.

The cervical spinal response (Field Potential at CV6) is simulated using a Dipole Field Model.

- Static Field Potential: This calculates the potential using a Source-Sink dipole model with a fixed conductivity (σe).
- Dynamic Current Pulse: The temporal evolution is governed by a current pulse function that models the rise and decay of the signal as the volley passes the cervical electrode.

Unlike the peripheral signals, the S1 Cortical response is derived from real human MEG (Magnetoencephalography) data.

- MNE Integration: The simulator loads a pre-recorded dataset from the “mne” library.
- Dynamic Interpolation: It interpolates and resamples the real MEG data to match the simulator’s specific time vector. Users can switch between different MEG channels (e.g., C3, C4) to see how the cortical SEP changes based on the electrode’s location.
- Signal Processing and Learning Integration:
- Averaging Engine: To mimic clinical practice, the simulator uses a FIFO (First-In-First-Out) buffer to perform signal averaging. This reduces injected Gaussian noise to reveal the evoked response.
- Waterfall Plotting: The system generates “Waterfall” views, which are historical stacks of averaged blocks, allowing users to monitor signal stability over time.
- Anomaly Injection and AI: The system includes an AnomalyInjector that can physically alter the signals (e.g., adding latency or decreasing amplitude) based on a JSON configuration. This is used for Learning Assessment, where the user must identify the problem and select the correct clinical action, which is then analyzed by an AI Agent for feedback.

### Appendix A.4. VEP Module

The Python code implements a VEP (Visual Evoked Potentials) Simulator designed for intraoperative monitoring (IOM) training. The application uses PySide6 for the user interface and pyqtgraph for high-speed signal rendering, simulating a two-channel recording from the left and right occipital hemispheres. The simulation employs a mathematical modeling approach to synthesize the typical waveforms recorded from the visual cortex in response to light stimuli.

Waveform Synthesis via Gaussian Summation: The core morphology of the VEP signal is generated by summing multiple Gaussian peaks. Each significant component of the VEP complex is defined by its latency, amplitude, and width (standard deviation) as follows:

- N75: A negative peak at ~75 ms.
- P100: The most clinically significant positive peak at ~100 ms.
- N145: A negative peak at ~145 ms.

The mathematical formula [13] used for each wave component is: f(t) = Amp⋅e−2σ2(t−Lat)2. This ensures a smooth, physiologically realistic curve rather than sharp, unnatural transitions.

Stimulus Modality and Hemispheric Distribution: The simulator accounts for the anatomical decussation of the visual pathway. It allows for three stimulation modes that dynamically scale the amplitudes across the two channels as follows:

- Bilateral: Full signal strength (100%) is applied to both the Left (Ch1) and Right (Ch2) Occipital channels.
- Monolateral (Left/Right): To simulate the partial crossing of fibers at the optic chiasm, the simulator applies full amplitude (1.0) to the contralateral hemisphere and a reduced scale factor (0.6) to the ipsilateral hemisphere.

Real-Time Averaging and Noise Injection: To replicate clinical recording conditions where the VEP signal is buried in background EEG noise, the following is conducted:

- Gaussian Noise: Every “sweep” (individual stimulus response) is injected with random Gaussian noise based on a user-adjustable standard deviation.
- Moving Average: The simulator calculates a continuous mean across sweeps. As the sweep count increases toward the target (e.g., 200 sweeps), the random noise cancels out, and the stable VEP waveform emerges from the baseline.
- Dynamic Simulation Speed: Users can adjust “Sweeps per update” to accelerate the averaging process for training purposes.

Educational and Anomaly Features: The code includes a framework for Learning Management. It can load JSON-based anomalies to modify signal parameters in real-time, for example, increasing the latency of the P100 wave to simulate clinical conditions like optic neuritis.

### Appendix A.5. CAP Module

The Python code implements a Multi-Distance CAP (Compound Action Potential) Simulator designed for intraoperative monitoring (IOM) training. It simulates the propagation of electrical signals [13] along a nerve trunk (such as the dorsal columns) and visualizes the recording at multiple anatomical sites. The application is built using a multithreaded architecture: a CAPWorker class handles the intensive mathematical modeling in a background thread, while the MultiDistanceCAPSimulator class manages the graphical user interface (GUI). The simulation methodology is based on a bottom-up biophysical model that synthesizes the global nerve response from individual fiber contributions [14,15].

Individual Fiber Modeling (SFAP): The “building block” of the simulation is the Single Fiber Action Potential (SFAP). The code provides the following two mathematical models for this:

- Bipolar Gaussian Model: This combines two Gaussian functions (one positive and one negative) to create the characteristic biphasic shape of an extracellular recording.
- Ricker Wavelet Model: This is an alternative mathematical model (often called the “Mexican Hat”) used to simulate the SFAP pulse.

Nerve Trunk Simulation (Temporal Dispersion): To simulate a compound potential, the code models a population of nerve fibers (default 100 to 100,000 fibers).

- Conduction Velocity (VC) Distribution: Each fiber is assigned a velocity within a specific range (typically 20.0 to 65.0 m/s).
- Spatial Propagation: The potential for a specific distance (d) is calculated by applying a time delay to each fiber (Delay = VCd).
- Compound Summation: The CAP displayed on the screen is the linear summation of all individual SFAPs. This naturally simulates temporal dispersion—as the recording site moves further from the stimulus, the signal becomes wider and lower in amplitude because faster and slower fibers arrive at increasingly different times.

Signal Conditioning and Averaging: Because the CAP is often buried in noise, the simulator replicates clinical recording techniques.

- Gaussian Noise Injection: Random noise is added to the raw signal to simulate background interference.
- FIFO Averaging: The simulator uses a First-In-First-Out (FIFO) buffer system (default 100 averages) to perform signal averaging, which improves the signal-to-noise ratio over time.
- Digital Filtering: Users can apply various filters in real-time, including Moving Average (Smooth), Spline Interpolation, and Butterworth Bandpass filters (e.g., 5–3000 Hz or 10–1500 Hz).
- Interactive Clinical Features:
- Velocity Measurement: The GUI includes interactive vertical cursors. By placing cursors on the peaks of signals from different distances (e.g., a wrist at 7 cm and an elbow at 20 cm), the software automatically calculates the measured conduction velocity in m/s.
- Anomaly Injection: The code integrates an AnomalyInjector that can physically alter the signal in real-time based on a JSON configuration. This is used for training scenarios where a student must identify clinical changes (such as ischemia or compression) and choose the correct corrective action from a dropdown menu.

### Appendix A.6. CMAP Module

The provided Python code implements a simulation framework for CMAP (Compound Muscle Action Potential) and MEP (Motor Evoked Potential) signals, which are critical components in intraoperative neuromonitoring (IOM) for assessing the integrity of motor pathways. The simulation is built on the principle of Summation of Motor Unit Action Potentials (MUAPs). Rather than using a single static wave, the code synthesizes a global muscle response by modeling hundreds of individual motor units and summing their electrical contributions.

Individual MUAP Waveform Modeling: The “building block” of the signal is the single MUAP and includes the following:

- Triphasic Morphology: The single_muap_waveform function models a triphasic (positive–negative–positive) wave. This is achieved by combining three Gaussian-like phases (P1, N1, and P2) with specific amplitudes and time offsets.
- Stochastic Variation: For every motor unit in the simulation, the code randomly varies the amplitude (simulating different motor unit sizes) and the duration/sigma (simulating the physical characteristics of the muscle fibers).

Conduction Velocity and Temporal Dispersion: A core feature of the CMAP simulation is how it handles the propagation of the signal from the nerve to the muscle.

- Conduction Velocity (VC) Distribution: Each motor unit is assigned a specific velocity based on a normal distribution (e.g., mean of 60 m/s with a standard deviation).
- Propagation Delay: The software calculates a unique delay for each unit based on the anatomical distance (Delay = VelocityDistance).
- Temporal Dispersion: Because different fibers conduct at different speeds, the individual MUAPs arrive at the recording electrode at slightly different times. This causes the resulting CMAP to “spread out” and lower in amplitude as the distance increases, accurately reflecting nerve physiology.

CMAP vs. MEP Simulation Logic: The code differentiates between a peripheral nerve response (CMAP) and a central nervous system response (MEP).

- CMAP Methodology: This primarily focuses on peripheral conduction velocity dispersion and a fixed synaptic/junctional delay (e.g., 2.3 ms) to model the time it takes for the signal to cross the neuromuscular junction.
- MEP Methodology: This introduces the concept of Central Jitter (tccm_spread_ms). Unlike the synchronized stimulation of a peripheral nerve, a cortical stimulation (tcMEP) results in desynchronized firing of the corticospinal tract. The code models this by adding a “jitter” or temporal spread to each MUAP, which creates the complex, multi-peaked morphology typical of clinical MEPs.

### Appendix A.7. EEG Module

The Python code implements an EEG (Electroencephalography) and Auxiliary Signal Simulator designed for real-time neurophysiological monitoring and processing. The application uses PySide6 for the interface and pyqtgraph for high-performance signal rendering, integrating the MNE-Python library for specialized EEG data handling. The simulation methodology is based on a template-based streaming model combined with dynamic artifact injection and real-time spectral analysis. The system acts as a “virtual amplifier” by streaming real EEG data from clinical files.

- MNE CNT Loading: The simulator loads Neuroscan .cnt files using the mne.io.read_raw_cnt module.
- Real-time Resampling: If the original file’s sampling frequency differs from the target (default 512 Hz), the code performs on-the-fly resampling.
- Microvolt Scaling: Data are scaled by a factor of −1 × 106 to ensure signals are displayed in standard microvolts (μV).

As the data are “played back” in chunks (default 100 ms intervals), several processing layers are applied, such as the following:

- Average Referencing: The spatial mean of all 16 EEG channels is subtracted from each channel to minimize common-mode noise.
- Frequency Filtering: The worker applies a bandpass filter (typically 1.6–35 Hz for EEG and 1.0–25 Hz for ECG) to remove slow drifts and high-frequency muscle interference.

To replicate the clinical environment, the add_eeg_artifacts_dynamic function overlays synthetic noise onto the clean EEG database on the user’s GUI controls:

- EOG Blinks: The simulator injects vertical eye movement artifacts into the frontal channels (FP1, FP2) by scaling the loaded EOG template.
- EMG Noise: High-frequency Gaussian noise is added to all channels to simulate patient tension or muscle movement.

The simulation extends beyond simple time-domain viewing through advanced background workers as follows:

- Block-based PSD: Every 2 s, the worker triggers a Power Spectral Density (PSD) calculation using the Welch method to display the frequency distribution (delta, theta, alpha, and beta bands).
- Spectral Whitening: A specific feature allows for 1/f “whitening” to flatten the power spectrum, making higher frequency oscillations like alpha and beta more visible against the dominant lower frequencies.
- ECG R-Peak Detection: If enabled, the system runs the analyze_r_peaks utility on the ECG channel, calculating the Heart Rate (BPM) and Heart Rate Variability (SDNN, RMSSD) in real-time.

Visualization Features: Sweep vs. Page Mode: The GUI supports “Sweep” (continuous scrolling) or “Page” (static screen updates) visualization modes.

Spectra Window: A separate window provides specialized views for channels PSD or Time-Frequency Representations (TFR), including an interactive cursor to measure specific decibel (dB) values at targeted frequencies.

### Appendix A.8. ECoG Module

The simulation does not generate purely synthetic mathematical data (unlike simpler simulators); instead, it employs a hybrid playback and artifact injection model.

Data Playback Engine:

- Template-Based Streaming: The simulator loads real ECoG recordings from .mat files (using scipy.io) and converts them into MNE RawArray objects.
- Worker-Timer Architecture: A dedicated ECoGWorker runs on a separate thread to prevent UI freezing. It streams data chunks at specific intervals (default 100 ms) to mimic live acquisition from an amplifier.
- Circular Buffer Logic: The _run_simulation_step method manages a circular pointer over the loaded dataset, ensuring continuous data flow by wrapping back to the start once the file ends.

Real-Time Signal Conditioning. As the data are streamed, several digital signal processing (DSP) steps are applied as follows:

- Common Average Reference (CAR): For each chunk, the spatial mean across all electrodes is subtracted from each channel to remove global noise and artifacts common to all sensors.
- Bandpass Filtering: A dynamic Butterworth filter (applied via scipy) allows users to adjust low-cut (e.g., 2 Hz) and high-cut (e.g., 300 Hz) frequencies in real-time to focus on specific oscillations like Gamma or Ripple bands.

JSON-Driven Artifact and Anomaly Injection: A core feature of this simulator is its ability to inject clinical or technical anomalies dynamically.

- ArtifactGenerator Integration: The simulator uses an external ArtifactGenerator class that reads configurations from a JSON file.
- Dynamic Modification: Based on the JSON “scenario,” the worker modifies the raw ECoG signal in real-time. This can include:
  - Specific Channel Targeting: Anomalies can be applied to all channels or a subset.
  - Time-Locked Events: Using a continuous time vector, the generator can overlay sinusoidal noise, spikes, or baseline drifts.

Advanced Feature—Persistent Pattern Search: The “Persistent Pattern Search Edition” includes a methodology to find recurring neurophysiological events (such as interictal spikes or specific oscillations).

- ROI Selection: Users select a “Region of Interest” on the plot by using a graphical UI element (LinearRegionItem).
- FFT-Based Cross-Correlation: To achieve a “100× speedup,” the code uses Fast Fourier Transform (via the fast_normalized_cross_correlation utility) to scan the entire recording for matches that exceed a user-defined correlation threshold (e.g., 0.80).

Averaging Logic: Once matches are found, the system extracts those segments and computes an Event-Related Average, allowing the user to compare the “Model” pattern against the statistical average of all found hits.

### Appendix A.9. Electromyography Free-Running Module

The EMG simulation module, which replicates signals recorded via subdermal needle electrodes, is modeled as a white noise signal that is overlaid with intraoperative anomalies, specifically A-trains (neurotonic), B-bursts (rhythmic), B-spikes (intermittent), and C-trains (complex). The module features an automated event-detection capability based on an adjustable amplitude threshold. All detected events and their associated annotations are logged and saved in a list detailing the timestamp and the identifier of the muscle exhibiting that specific pattern.

### Appendix A.10. Anesthesia Module

The Python code implements an anesthesia simulator tailored for intraoperative monitoring (IOM) contexts. It simulates the pharmacokinetics (PK) and pharmacodynamics (PD) of common anesthetic agents and their impact on vital signs like Heart Rate (HR), Blood Pressure (MAP), and Heart Rate Variability (HRV). The simulation methodology is based on three main pillars of target-controlled infusion (TCI) models, pharmacodynamic interaction logic, and stochastic vital sign generation.

The simulator calculates plasma concentrations (Cp) and effect-site concentrations (Ce) in real-time using the following established medical models:

- Propofol (Schnider Model) [16]: Uses a three-compartment model to calculate how Propofol distributes and is eliminated based on the patient’s age and weight.
- Remifentanil (Minto Model) [17]: A specialized model for high-potency opioids, accounting for rapid onset and offset.
- Ketamine: Modeled as a sympathomimetic agent that counteracts the depressive effects of other drugs on the cardiovascular system.
- Sevoflurane: Simulates inhalation anesthesia measured in MAC (Minimum Alveolar Concentration).

Pharmacodynamic (PD) Interaction Logic: The code simulates how these drugs interact to affect the “Patient State”.

- Synergy vs. Antagonism: This calculates a global depressive effect from Propofol, Remifentanil, and Sevo. This combined effect reduces the Heart Rate and MAP.
- Ketamine Offset: Ketamine is programmed with a “sympathomimetic” factor. As the Ketamine concentration increases, it mathematically offsets the bradycardia and hypotension caused by Propofol, raising the HR and MAP back toward baseline.

Vital Signs Simulation Engine. The physiological signals are generated using a combination of the following deterministic and stochastic processes:

- ECG Waveform: A synthetic ECG signal is generated by concatenating a baseline with a “QRS complex” (a high-frequency triangle wave). The frequency of these complexes is tied directly to the calculated instantaneous Heart Rate.
- Respiratory Sinus Arrhythmia (RSA): The simulator introduces small, natural fluctuations in Heart Rate to mimic the interaction between breathing and the heart.
- HRV Metrics: The code calculates SDNN (standard deviation of NN intervals) and RMSSD in real-time. These metrics decrease as the depth of anesthesia increases, providing a neurophysiological indicator of autonomic nervous system depression.
- Interactive Clinical Features:
- MAP Calculation: The simulator approximates the Mean Arterial Pressure (MAP) using the drug concentrations and a random walk component to simulate blood pressure stability.
- Real-time Controls: Users can adjust drug dosages via sliders, and the simulation immediately updates the decay curves (Ce) and the resulting physiological waveforms.
- Visualization: This uses pyqtgraph to provide a “Vital Signs Monitor” view (ECG) alongside a “Pharmacokinetic” view (Concentration curves), bridging the gap between drug administration and physiological response.

## Appendix B

Simulation systems can be classified into the following three fundamental categories:

**Biophysical Simulators:** Tools that model biophysical principles using a bottom-up approach, starting from individual ion channels and single neurons. Their primary objective is to foster conceptual understanding of fundamental biological mechanisms.

**Clinical Waveform Generators:** Instruments engineered to replicate clinically recognizable signals and their pathological variants. These tools are specifically designed for training in diagnostic pattern recognition.

**Complex Systems and Research Platforms:** High-level frameworks for modeling large-scale neural networks and global brain dynamics. These platforms often necessitate advanced programming skills and are primarily suited for academic research or advanced postgraduate medical education.

**Table A1:** Comparison of simulation tools: Pros and Cons vs. ION-Sim.

CONS (Limitations Compared to ION-Sim)	PROS (Advantages of the Alternative)	Examples	Category/Concept
Hardware-bound; lacks a standalone, offline simulation mode for training without a patient or expensive equipment.	Provides exact training on industry-specific software interfaces; clinical-grade diagnostic utility.	Cadwell Sierra, Inomed, Neurosoft	**Commercial Proprietary Solutions**
Focuses on microscopic/cellular mechanisms rather than macroscopic, multimodal clinical intraoperative monitoring scenarios.	Excellent for teaching foundational, cellular-level neurophysiology (ion channels, resting/action potentials) without programming skills.	Neurosim 5, MetaNeuron	**Biophysical Simulators**
Requires advanced expertise; focuses on macroscopic brain rhythms rather than generating specific, real-time surgical monitoring signals (SSEP/MEP).	Highly advanced for modeling large-scale, whole-brain network dynamics using personalized neuroimaging data.	The Virtual Brain (TVB)	**Complex Systems and Brain Research**
Requires advanced programming skills (MATLAB); engineering-focused rather than tailored for clinical intraoperative pattern recognition.	Open-source; provides a known ground truth for validating advanced signal processing and BCI algorithms.	SEREEGA	**EEG/ERP Simulation Frameworks**
Requires expensive proprietary software; engineering-oriented rather than being driven by a clinical Learning Manager and surgical scenarios.	Robust, programmable control over basic signal parameters and real-world artifacts (e.g., 50/60 Hz powerline noise).	LabVIEW Biomedical Toolkit	**Engineering and Signal Toolkits**
Highly specialized to single modalities (EMG/MNCS); lacks the unified, multimodal intraoperative scope (SSEP, VEP, BAEP, MEP) of ION-Sim.	Exceptional biophysical fidelity for specific modalities (EMG, MUAPs) with explicit modeling of pathological neuromuscular diseases.	EMG Simulator by Stålberg Software	**Diagnostic Waveform Generators**
Highly dependent on expensive physical lab hardware; designed for basic physiology rather than software-only surgical simulation.	Excellent for hands-on physiological lab sessions utilizing pre-configured experiments and biological signal acquisition.	ADInstruments LabChart, iWorx LabScribe	**Hardware-Integrated Educational Ecosystems**
Hardware-dependent and tailored strictly to a single surgical procedure, lacking the versatile, generalized software framework of ION-Sim.	Offers high physical realism by combining spatial/magnetic tracking with tangible anatomical models.	Mastoidectomy training system	**Procedure-Specific and Hybrid Systems**
Predominantly focused on manual skills rather than the cognitive analysis and interpretation of neurophysiological traces.	Excellent for training manual dexterity, haptic feedback, and anatomical spatial navigation.	SurgeonsLab, InSimo	**Surgical Procedural Simulators**

**Neurosim 5:** This is a commercial software package specifically engineered for teaching cellular neurophysiology at both undergraduate and postgraduate levels. Its core philosophy centers on accessibility; it requires no prior programming knowledge, enabling students to conduct virtual experiments that would be unfeasible in a traditional laboratory setting [18]. The strength of the platform lies in its modular architecture, which deconstructs the complexities of neurophysiology into several distinct components:

**GOLDMAN Module:** Simulates the Goldman–Hodgkin–Katz equation, allowing students to manipulate ionic concentrations and membrane permeabilities to directly observe the generation of the resting potential. This module provides a tangible representation of the Nernst and Goldman equations, which are fundamental to all aspects of electrophysiology [19].

**HODGKIN–HUXLEY Module:** Implements the original action potential model. Students can apply stimuli in current-clamp or voltage-clamp modes, vary the temperature, administer virtual pharmacological agents (such as tetrodotoxin to block sodium channels), and investigate phenomena such as refractory periods and accommodation [19].

**PASSIVE CONDUCTION Module:** Simulates the cable properties of an ideal axon or dendrite. This is crucial for grasping electronic conduction and signal propagation—key concepts for understanding temporal dispersion in evoked potentials and nerve conduction studies [20].

**NETWORK Module:** Facilitates the construction of small neuronal circuits. Students can link neurons via chemical or electrical synapses, exploring emergent phenomena such as lateral inhibition, oscillatory circuits, and synaptic integration. This module serves as a conceptual bridge toward understanding the network activity that underlies EEG signals [18].

Furthermore, this platform is supported by an extensive suite of tutorials, guided exercises, and classroom activities, offering a “turnkey” solution for educators [18]. Its longevity and continuous updates are well-documented in academic literature, consolidating its status as a benchmark pedagogical tool [21].

**MetaNeuron:** A freeware application designed for interactive educational sessions, focusing on core neurophysiological principles through simplified simulations [22]. Its interface facilitates real-time parameter manipulation, providing instantaneous feedback on neuronal responses and promoting an exploration-based learning paradigm. Its guided “lessons” cover topics parallel to those in Neurosim, including resting membrane potential (based on passive conductances and membrane time/space constants) and the action potential as modeled by the Hodgkin–Huxley equations [22]. Its open-access nature makes it an ideal solution for resource-constrained educational settings.

**SEREEGA (Simulating Event-Related EEG Activity):** An open-source MATLAB-based toolbox engineered for generating simulated EEG data, with a specific focus on event-related potential [23]. Its primary objective is to provide researchers with a known ground truth to validate signal processing methods, source localization techniques, or Brain–Computer Interface (BCI) classification algorithms [24]. SEREEGA is an optimal tool for advanced curricula in biomedical engineering, computational neuroscience, or signal processing. It enables students to gain hands-on experience with the EEG forward problem: how dipolar source activations within the brain volume project onto scalp electrodes. Users can define components with specific spatial coordinates and orientations, assign various activation signals (such as Event-Related Potentials (ERP) or Event-Related Spectral Perturbations (ERSP)), and observe the resulting EEG [23]. While the software supports diverse head models and offers high extensibility, it requires a MATLAB environment and familiarity with the EEGLAB toolbox [23].

**The Virtual Brain (TVB):** A high-performance open-source research platform designed to simulate large-scale, whole-brain network dynamics [25]. Unlike other simulators, TVB constructs personalized brain models integrated with an individual’s structural neuroimaging data (e.g., DTI/DSI tractography for white matter connectivity). Using this connectivity matrix, it simulates neuronal population activity to generate emergent neurophysiological signals, including EEG, MEG, and fMRI [25]. In a pedagogical context, TVB is best suited for postgraduate and research levels. It offers unparalleled insights into how brain anatomy constrains and shapes dynamic function, allowing for the study of emergent brain rhythms or the propagation of pathological activity, such as epileptic seizures [9]. The TVB EduPack, along with numerous online tutorials, provides educational use cases, interactive Jupyter notebooks, and video guides that lead users through the replication of published studies on epilepsy and Alzheimer’s disease [26].

**LabVIEW Biomedical Toolkit:** An add-on for National Instruments’ LabVIEW graphical development environment, a standard in engineering for data acquisition and analysis [27]. Within this toolkit, the “Simulate EEG” Virtual Instrument (VI) offers a pragmatic and highly controllable approach to EEG signal simulation [28]. Through a graphical user interface (GUI), users can specify fundamental signal parameters, including output amplitude, sampling frequency, and the relative power of canonical frequency bands (delta, theta, alpha, and beta) [28]. A key pedagogical feature is the ability to introduce realistic artifacts; users can inject controlled-amplitude white noise and, crucially, 50 Hz or 60 Hz powerline interference to simulate real-world recording conditions [28].

**EMG Simulator by Stålberg Software:** Widely considered the benchmark tool for EMG education and research [29]. This standalone Windows application is independent of specific EMG hardware [29]. Its exceptional fidelity stems from its underlying biophysical model: the software generates Motor Unit Action Potentials (MUAPs) by summating individual muscle fiber action potentials, calculated according to current literature and biophysical principles [29]. Users maintain granular control over a wide range of physiological and recording parameters, including:

Fiber parameters: Diameter and jitter (variability in neuromuscular transmission).

Motor unit parameters: Fiber count, spatial distribution, and end-plate localization. Recording parameters: Electrode type (e.g., concentric) and precise intramuscular positioning [29].

This level of customization allows for an in-depth exploration of how MUAP morphology (amplitude, duration, and phases) depends on these factors. However, its most powerful pedagogical feature is the capacity to explicitly simulate pathological conditions. Users can model scenarios for myopathic diseases (fiber loss), neurogenic disorders (reinnervation with increased fiber density), and myasthenic syndromes (increased jitter and blocking) [29]. This functionality is indispensable for training neurologists and clinical neurophysiologists, as it correlates pathophysiological findings with diagnostic EMG patterns in a controlled environment. The developer also provides the MNCS Simulator, designed to teach the principles of motor nerve conduction studies, including complex concepts such as conduction block and axonal loss [30].

**LabVIEW Biomedical Toolkit:** Analogous to its EEG counterpart, the toolkit includes a “Simulate EMG” VI. This instrument allows for the configuration of parameters such as contraction time, amplitude, noise levels, and frequency spectra [31]. Although less biophysically detailed than the Stålberg simulator, it provides a robust, engineering-oriented platform for understanding the fundamental properties of both surface and intramuscular EMG signals. To date, no standalone software equivalent to Neurosim or the Stålberg EMG Simulator has been identified that is specifically designed to teach the generation and clinical interpretation of SEPs, VEPs, BAEPs, or MEPs [32].

**ADInstruments LabChart:** This platform constitutes an integrated hardware (PowerLab) and software (LabChart) ecosystem for biological signal acquisition [33]. It utilizes various stimulators (electrical, visual, auditory) to evoke real potentials from human or animal subjects [34]. Its pedagogical value lies in its pre-configured experimental packages and sample data files. For instance, the “Visual Evoked Potential (VEP)–Pre-Lab Prep” module [35] enables students to analyze VEP data, teaching them to measure the latencies and amplitudes of key components (e.g., N75, P100, N145) and interpret their clinical significance, even in the absence of live recordings [36].

**iWorx LabScribe:** Similar to LabChart, iWorx provides an integrated hardware-software system tailored for educational laboratories [37]. The LabScribe software includes modules for Event-Related Potential (ERP) analysis and offers guided experiments covering auditory and visual reflexes, providing a framework for studying evoked potentials. While the platform includes an ECG simulator and an arrhythmia generator—demonstrating significant simulation capabilities—these do not currently extend to neurophysiological-evoked potentials [38].

**Cadwell Sierra Software:** This is a clinical-grade diagnostic system used in hospital settings for EMG, nerve conduction studies, and a comprehensive suite of evoked potentials (SSEP, VEP, AEP, and MEP) [6]. Its application in education occurs primarily through hands-on clinical workshops, where participants utilize the hardware to perform tests and analyze clinical cases [5]. It does not appear to feature a standalone educational “simulation mode” that can be operated without a patient or subject.

Scientific literature regarding **MEP simulation** focuses largely on the creation of statistical models to understand inter-trial variability—a research goal aimed at optimizing Transcranial Magnetic Stimulation (TMS) dosing [39]. These models, however, are not implemented in accessible educational software. Clinical systems, such as the Cadwell Sierra, provide protocols for MEP acquisition but lack dedicated simulation features for teaching purposes [40].

This “evoked potential simulation gap” creates a pedagogical dependency on expensive hardware ecosystems or advanced technical expertise (such as MATLAB proficiency for SEREEGA). This introduces a significant barrier to accessibility and equity for institutions lacking substantial laboratory budgets or dedicated technical staff. While foundational concepts can be taught using free or low-cost software, the practical instruction of evoked potentials risks becoming a privilege reserved for well-funded institutions capable of maintaining high-end data acquisition labs. Consequently, the development of a user-friendly, open-source evoked potential simulator—analogous to Neurosim—would represent a substantial contribution toward the global democratization of neurophysiological education.

## Appendix C

We present two examples of realistic signal recordings obtained in a surgical scenario. Naturally, the signals featured in the various simulation modules differ to some extent to ensure clarity and facilitate pedagogical simplification.
